# The Potential of Anti-Bullying Efforts to Prevent Academic Failure and Youth Crime. A Case Using the Olweus Bullying Prevention Program (OBPP)

**DOI:** 10.1007/s11121-021-01254-3

**Published:** 2021-05-16

**Authors:** Nicolai Topstad Borgen, Dan Olweus, Lars Johannessen Kirkebøen, Kyrre Breivik, Mona Elin Solberg, Ivar Frønes, Donna Cross, Oddbjørn Raaum

**Affiliations:** 1 Department of Sociology and Human Geography, University of Oslo, Oslo, Norway; 2 Department of Special Needs Education, University of Oslo, Oslo, Norway; 3 Department of Health Promotion and Development, University of Bergen, Bergen, Norway; 4 Statistics Norway, Oslo, Norway; 5 Regional Centre for Child and Youth Mental Health and Child Welfare, NORCE, Bergen, Norway; 6 Norwegian Center for Child Behavioral Development, Oslo, Norway; 7 University of Western Australia, Telethon Kids Institute, Perth, Australia; 8 Ragnar Frisch Centre for Economic Research, Oslo, Norway

**Keywords:** Bullying prevention programs, Olweus, Crime prevention, Academic failure

## Abstract

The effectiveness of bullying prevention programs has led to expectations that these programs could have effects beyond their primary goals. By reducing the number of victims and perpetrators and the harm experienced by those affected, programs may have longer-term effects on individual school performance and prevent crime. In this paper, we use Norwegian register data to study the long-term impact of the Olweus Bullying Prevention Program (OBPP) on academic performance, high school dropout, and youth crime for the average student, which we call population-level effects. The OBPP program is widely acknowledged as one of the most successful programs reducing school-level bullying; yet, using a difference-in-difference design, no statistically significant population-level effects of the OBPP were found on any of the long-term outcomes in this study. When studied at the population level, as in the current project, the base rate prevalence of bullying is a major explanatory factor for these results. Earlier studies have shown that OBPP reduces bullying prevalence by 30–50%. This decrease translates into absolute reductions in bullying victimization and perpetration at the population level of “only” four and two percentage points, respectively. Our results suggest the average causal effects of school bullying involvement are too small to translate this reduction in bullying into a sizeable population-level impact on students’ long-term outcomes. However, a limited potential of anti-bullying programs to prevent *population-*level adversity can very well be compatible with substantial program effects for *individual* bullies and victims. Further, our results do not speak to the main objective of anti-bullying programs of limiting childhood abuse and safeguarding children’s human rights.

**Supplementary Information:**

The online version contains supplementary material available at 10.1007/s11121-021-01254-3.

Exposure to bullying by peers in schools is associated with and very likely is a direct cause of often incapacitating psychological, social, and academic problems in targeted students (Arseneault, 2018; Klomek et al., 2015; Nakamoto & Schwartz, 2010; Olweus, 1993; Olweus & Breivik, 2014). Perpetrators develop problems as well but usually of a more externalizing nature, such as anti-social behavior, criminality, and drug use (Cook et al., 2010; Klomek et al., 2015; Ttofi & Farrington, 2008). These adverse effects have stimulated the development and testing of anti-bullying programs (Smith, 2019). While these bullying prevention programs’ effectiveness is shown to vary considerably, on average, they decrease both school bullying and victimization by about 20% (Gaffney et al., 2019a, b; Ttofi & Farrington, 2011). From these promising evaluations, some have suggested that anti-bullying programs can *also* be used to prevent academic failure (Cornell et al., 2013), early crime, suicide, and mental health problems (Klomek et al., 2015; Menesini & Salmivalli, 2017; Ttofi et al., , 2011, 2012). In effect, anti-bullying programs may be considered early interventions for public health (Gaffney et al., 2019a, b; Menesini & Salmivalli, 2017).

However, sizeable population-level effects of bullying prevention on long-term outcomes are only likely if programs substantially reduce the fraction of the student population who are bullies and/or victims and the causal influences of bullying are large. Even if few studies question the negative impacts of bullying, some doubt its effects are substantial (Schoeler et al., 2018; Silberg & Kendler, 2017). If the causal influence of bullying is exaggerated, then the potential gains from interventions are overestimated, as even eliminating bullying may result in only minor improvements in outcomes such as academic failure and criminal charges (Schoeler et al., 2018; Silberg & Kendler, 2017). Conversely, the population-level gains from bullying prevention are limited if few students escape bullying due to the program, even if the causal effect of bullying is large. We are aware of no studies that have tested whether any long-term protective impact of bullying prevention efforts is supported by the data.

Against this backdrop, the present paper fills a gap in the literature by examining the long-term effects for students exposed to the Olweus Bullying Prevention Program (OBPP). We compare the long-term outcomes of students from OBPP schools with earlier cohorts of students who went to the same schools (who were not exposed to any bullying prevention programs). Long-term outcomes were defined as student outcomes realized *after* they have graduated. Using a difference-in-difference (DiD) design, we investigate whether attending an OBPP school (ages 9–13) affects academic grades (age 16), high school dropout (by age 21), and youth crime (by age 20) by means of full-population Norwegian register data.

Our point of departure is that, unlike many anti-bullying programs with minimal impact on bullying (Smith et al., 2012; Swearer et al., 2010), programs inspired by the work of Dan Olweus, and especially the OBPP program, have been highlighted to be effective at reducing bullying (Ttofi & Farrington, 2011). Thus, the OBPP in Norway provides an opportunity to test the scope of anti-bullying programs to prevent adverse long-term outcomes. As part of the general intervention strategy of the OBPP, however, bullies and victims are not identified in the data (aggregated school-level bullying data were used). Hence, we cannot study the long-term effects of bullying prevention for potential victims and bullies in this study. Instead, we are restricted to studying average program effects across all students, which we call population-level program effects.

The long-term effects of a universal or selective school-based anti-bullying program are different and can be studied in distinct ways. One approach uses the school as the basic analytic unit. As a recent example, co-authors of the current article followed up some 200 schools that had successfully implemented the universal OBPP program in the 2001–2005 period (Olweus et al., 2020). The study found that schools that seemed to continue according to the principles of the program had a better long-term development with regard to bullying problems than initially equally successful schools that appeared to have stopped using the program in the 2–8 years after original implementation. The value of such long-term school-level effects lies in the fact that consecutive cohorts of students will attend schools with reduced levels of bullying problems and thereby have a lower risk of being exposed. In the current project, we study outcomes of students who, at an earlier point in time, have been exposed to OBPP in school and compare them with similar students not exposed to OBPP. Our study seeks to find out if bullying-involved students who may have benefited from the program in the short-term have retained some or all of such effects after a specified time.

The expected long-term effects of anti-bullying programs are derived from research that evaluates the effectiveness of anti-bullying efforts and distinct research that identifies the long-term consequences of bullying (Cornell et al., 2013; Gaffney et al., 2019a, b; Klomek et al., 2015; Menesini & Salmivalli, 2017; Ttofi et al., 2011). Evaluation of bullying prevention programs has mostly focused on the main objective of reducing bullying and rarely studied secondary prevention effects. Some exceptions include studies of the KiVa program in Finland that found some secondary effects on depression, self-esteem, and anxiety (Juvonen et al., 2016; Williford et al., 2012). Secondary prevention effects have also been indicated for OBPP on classroom social climate and anti-social behavior, such as vandalism and theft in Norway (Olweus, 1991) and the USA (Limber et al., 2004). An early study in the Netherlands on a bullying prevention program inspired by OBPP indicated effects on depression but found no effects on delinquent behavior, school wellbeing, or psychosomatic complaints (Fekkes et al., 2006).

## Bullying Prevention and Potential Long-Term Effects

### The Effectiveness of Bullying Prevention

Bullying is defined as repeated aggressive behavior, carried out by a group or an individual, with the intent to inflict injury or discomfort, and is characterized by intentionality, repetitiveness, and power imbalance (Olweus, 1993). The prevalence of bullying varies widely by definition and measurement, age group, and country. Among school-age children in many Western countries, about 4–9% frequently engage in bullying, and 9–25% are bullied. The prevalence might be considerably higher in certain other non-Western countries (Menesini & Salmivalli, 2017).

While bullying was once considered a normative behavior of childhood (Silberg & Kendler, 2017), it is now recognized as a major problem with serious implications for bullies and/or victims (Olweus, 2013; Swearer et al., 2010). The first efforts to prevent bullying in schools date back to 1983, which was later known as the Olweus Bullying Prevention Program (OBPP) (Olweus, 1991, 1993, 2013). While several different bullying prevention programs have been developed since then, the OBPP remains one of the most researched and one of the most effective at reducing bullying worldwide (Gaffney et al., 2019a, b; Olweus & Limber, 2010; Ttofi & Farrington, 2011).

The OBPP is a whole-school or universal anti-bullying program with targeted components designed to reduce the prevalence of existing bullying problems, prevent the development of new problems, improve peer relations at the school, and build a sense of community (Olweus, 2013; Olweus & Limber, 2010). The OBPP is built on principles derived from an authoritative conceptual framework, which have been translated into several coordinated components, implemented at the school, classroom, individual, and (in the USA in particular) community levels. School personnel works to restructure schools’ social environment in order to reduce opportunities and rewards for bullying and build a sense of community.

In the most comprehensive meta-analysis to date, covering 100 program evaluations, Gaffney et al. (2019a) found that while the variability of program effects are large, on average, anti-bullying programs decrease bullying perpetration and bullying victimization by about 20% and 15%, respectively. The OBPP program has been shown to reduce bullying problems by about 30–50% in elementary school after 8 months of implementation in Norway (Olweus & Limber, 2010), with somewhat weaker effects in the USA (Limber et al., 2018).

### Formalization of Potential Long-Term Effects of Bullying Prevention

**Fig. 1:**
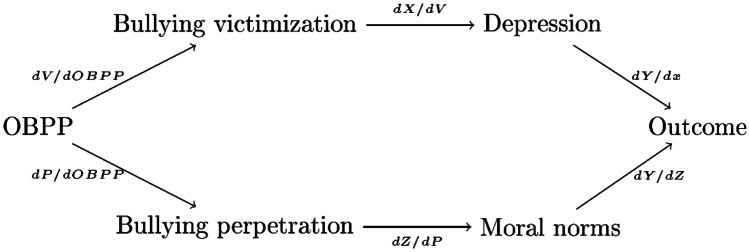
Illustration of potential program effects on long-term outcomes. We can decompose the association between OBPP and the outcome as: $\frac{dY}{dOBPP}=\frac{dV}{dOBPP}\frac{dX}{dV}\frac{dY}{dX}+\frac{dP}{dOBPP}\frac{dZ}{dP}\frac{dY}{dZ}$

The potential intervention effects at the population level are closely related to the effectiveness of bullying prevention and the consequences of fewer students being directly involved in bullying as victims or perpetrators. We illustrate this in Fig. 1, where *Y* is student long-term outcome, *V* is victim probability, *P* is the probability of being a perpetrator, and, finally, *X* and *Z* are factors mediating the link between bullying and the outcome for victims and bullies, respectively. In this conceptual framework, we can interpret the variables as student averages at the school level, in accordance with the empirical evidence presented in the Results section later.

Importantly, when examining program effects at the school level, we ask whether the proportion of students who experience adverse outcomes diminishes because of the intervention. Thus, we are *not* specifically identifying the long-term intervention effects for students involved in bullying as victims or bullies. Even so, most of the intervention effects are likely to operate through consequences for students with a high likelihood of involvement in bullying as victims or perpetrators. Thus, the overall program effect depends largely on the combined implications of less bullying victimization ($dV/dOBPP$) and fewer bullies ($dP/dOBPP$). Focusing on the victims, the lower frequency of bullying affects long-term outcomes if a lower victimization rate leads to a lower frequency of mental health problems or other mediators ($dX/dV$). The program effect on the long-term outcome also hinges on the magnitude of mediator effects on the outcome ($dY/dX$). The same logic applies to any long-term effect that operates through the number of bullies, but the mediators are likely to be different.

It has also been suggested that anti-bullying programs may positively affect outcomes via reductions in witnessed bullying, which has been linked to heightened anxiety, school dislike, substance abuse, and poorer mental health (Salmivalli et al., 2011). Finally, the program may influence students’ long-term outcomes via a better learning environment and higher teacher presence (Pyhältö et al., 2015), benefitting all students. Such “externalities” will be in addition to those achieved by reducing the number of victims and perpetrators.

Since the effect of bullying prevention on a long-term outcome is a product of several causal chains, the population-level effect is likely to be small and hard to identify empirically. First, and most importantly, the overwhelming majority of students is not involved in bullying and is therefore largely unaffected by the program. In Norway, substantial reductions in bullying problems by 30–50% after working with OBPP translate into an absolute change in victims and bullies of “only” 4 and 2 percentage points since the initial prevalence of bullying is low. Second, for students involved in bullying, long-term causal effects on academic and behavioral outcomes are likely to be rather small, given, for example, the time span between the events in the chain may dilute the effects. (In our study, the bullying is reduced at age 13, while outcomes are long-term at age 16 to 21.) Nevertheless, even small population-level intervention effects may be cost-beneficial from a public health and policy perspective because the economic and social burden of academic failure and criminal charges is substantial (Cohen & Piquero, 2009; Levin & Belfield, 2007).

### Evidence of the Long-Term Effects of Victimization and Perpetration

Possible long-term effects of the OBPP conducted at the elementary level (grades 4–7) must be mediated by some intermediate variable(s), and the mediating variable(s) may differ depending on the type of outcome variable and presumably differ for bullying perpetration and victimization. Bullying is a form of aggressive behavior, and bullies score high on dimensions of anti-social behavior as early as elementary school (Cook et al., 2010; Solberg & Olweus, 2003). Thus, by not bullying others, the potential perpetrator may be less likely to participate in gangs, spend less time with anti-social friends, and concentrate more on learning in school. Further, children involved in bullying others may experience weakening school bonding, resulting in a cycle of alienation leading to intensified aggressive behavior (Ttofi & Farrington, 2008; Ttofi et al., 2012). In contrast, pure victims are typically cautious, sensitive students with elevated levels of internalizing symptoms such as anxiety and depressive tendencies (Cook et al., 2010; Olweus, 1993) and are not expected to develop anti-social behavior.

Concerning *anti-social behavior*, evidence from 19 studies indicates that bullying other students in school is highly associated with an increased risk of elevated levels of aggression and violence several years after the school bullying occurred (Ttofi et al., 2011, 2012), although the causal nature of these associations is unclear. Compared to the effects of bullying perpetration, the impact of bullying victimization on anti-social behavior is expected to be small, as confirmed by Ttofi et al. (2012). Moreover, a recent meta-analysis by Schoeler et al. (2018), which used more stringent criteria for inclusion, found a lower association between bullying victimization and externalizing symptoms (including delinquency and conduct problems) than Ttofi et al. (2012). However, none of the studies above separated pure victims from bully-victims. Consistent with Klomek et al. (2015) findings reporting differential effects for pure victims and bully-victims, it can be assumed bully-victims account for most – but probably not all (Arseneault et al., 2006) – of the anti-social activities contributing to the slightly elevated risk of anti-social behavior for bullying victimization.

In this study, two of the outcome variables concern the relationship between academic achievement and bullying victimization and perpetration. In a meta-analysis of 33 studies, Nakamoto and Schwartz (2010) found a small negative correlation of −0.12 between bully victimization and academic achievement. The negative relationship is interpreted as a result of underlying and/or mediating variables such as poor emotional regulation, depressive symptoms, and decreased academic engagement. The weak short-term relationship between bullying victimization and academic achievement was confirmed more recently by Schoeler et al. (2018). A meta-analysis by Cook et al. (2010) shows that bully-victims and, to a lesser extent, bullies have lower academic achievement compared to pure victims, suggesting the effects on achievement may be stronger for bullies and bully-victims than for pure victims.

## Methods

### Register Data and Variables

The main data source in this paper is population-wide register data, covering all students born between 1980 and 1999 who attended an elementary school (grades 1–7), excluding children of immigrants who arrived in Norway after school starting age.^1^ Compulsory education in Norway begins at the age of six and lasts for 10 years, with primary education in grades 1 to 7 and lower secondary education in grades 8 to 10. Few students receive compulsory education in private schools (about 4%), and all schools are publicly funded. There are three main types of schools in Norway: elementary school (grades 1–7), lower secondary school (grades 8–10), and combined primary and lower secondary schools (grades 1–10). In the current project, the target population is elementary schools with grades 1–7, which is the largest group of schools in Norway.

Data from all 225 elementary schools that started implementing OBPP between fall 2001 and spring 2004 were used. The schools represent six different cohorts beginning implementation at half-year intervals, with 52 schools starting implementation in fall 2001, 43 schools in spring 2002, 55 schools in fall 2002, 116 schools in spring 2003, 13 schools in fall 2003, and, finally, 38 schools in spring 2004. Of these 225 elementary schools, 17 schools were excluded because they had various problems during the implementation period, including school closure, major restructuring (e.g., merger and extension of grade structure), or they conducted only one of the two surveys that were a criterion for being included. Robustness checks in Appendix Fig. C10 show that including the 17 schools does not influence the results notably. In the final analysis sample, the data from 208 program schools and 1483 control schools that did not implement the OBPP were used. See Online Appendix Table C1 and Online Appendix Fig. C1 for details describing when schools implemented OBPP.

**Table 1:** Descriptive statistics

	*N*	Mean	SD	Min	Max
Completed upp. sec. educ. by age 21	531,277	0.717	0.451	0	1
Examination grades at age 16	526,271	37.971	10.113	0	60
Charged ever by age 20	531,277	0.176	0.381	0	1
Years before/after OBPP					
Prior to implementation (−4 to −1)	719,262	0.032	0.177	0	1
Exposed for 1 year	719,262	0.009	0.094	0	1
Exposed for 2 years	719,262	0.009	0.095	0	1
Exposed for 3 years	719,262	0.009	0.094	0	1
Exposed for 4 years	719,262	0.009	0.095	0	1
Control schools	719,262	0.932	0.252	0	1
Parents’ education	718,549	4.762	1.557	0	8
Parents’ earnings (in 1000 NOK)	719,246	390.687	199.677	0	2557
Gender	719,262	0.487	0.500	0	1
Immigrant background	719,262	0.033	0.179	0	1
Year of birth	719,262	1990.113	5.518	1980	1999

Individual control variables are coded as year of birth (dummies), gender (girl = 1), average of father’s earnings at age 11–15 (linear and quadratic term), average of mother’s earnings at age 11–15 (linear and quadratic term), father’s educational level (9 dummies for primary or lower education, lower secondary education, some upper secondary education, completed upper secondary education, post-secondary non-tertiary education, undergraduate level, graduate level, post-graduate level, unspecified), mother’s educational level (9 dummies, same as father’s educational level), and immigrant background (5 separate dummies for immigrated, born in Norway to immigrant parents, foreign-born with one native parent, born in Norway with one foreign-born parent, and foreign-born to Norwegian-born parents)

The treatment indicator tracks the position of each school grade cohort relative to the year of program implementation, from 4 years before the program implementation to 4 years after. Students are labeled −1 if they finished elementary school either the same spring as the baseline was completed or if the baseline was completed in the following fall. The next cohort, exposed to OBPP for either the entire 7th grade (if baseline during the spring semester) or the spring semester of 7th grade (if baseline during the fall semester), is labeled 1. Cohort 4 is the first cohort exposed to the program through grades 4–7. Since exposure equals time since implementation, any differential effects across the cohort will capture the joint impact of length of exposure for the individual and length of implementation at the school.

Three main outcome variables were used in the analyses. Examination grades is the externally and anonymously graded score at the end of lower secondary school (grade 10 at age 16), standardized with a zero mean and standard deviation of one. The completion of upper secondary education is measured at age 21. Criminal charge measures whether students have ever been charged for a criminal act by age 20, including both felony offenses and misdemeanor. The Online Appendix shows results for different age cutoffs, felony offenses and misdemeanor separately, and for detailed type of criminal charge (Appendix Figs. C6, C7, and C8).

Individual control variables are year of birth, gender, parental earnings, parental education, age, and immigrant background. Descriptive statistics of the outcome, treatment, and controls can be found in Table 1.

### Linking Students to Schools in Register Data

Norway does not maintain a registry of the primary school students attend. To link students to schools, we impute the most likely school attended from students’ residential addresses (for details, see Appendix A). This imputation will cause some misclassification of the school attended and thus of program exposure; some students will be incorrectly classified as exposed or not exposed to the program. This type of misclassification could bias the effect estimates either upwards or downwards; however, this study’s misclassification will probably cause a slight attenuation bias in the effect estimates. Assuming it is conditionally random, we argue in Appendix A that the effect estimates are attenuated by a factor of about 0.9, and we can inflate coefficients and standard errors by about 11% (1/0.9 = 1.11) to adjust for the bias. This adjustment is relatively minor, and we will not explicitly implement this adjustment as part of our estimator, but instead refer to it in our discussion of the results.

### Analytic Approach to Study Long-Term Effects of OBPP

Schools choosing to implement a program like OBPP may differ from other schools (e.g., higher levels of bullying); hence, we cannot directly compare students from program and control schools. Our strategy is to compare outcomes of different birth cohorts *within the same school* and use a difference-in-difference (DiD) model that accounts for selection into treatment as well as time/cohort effects common to all schools. The DiD model compares outcomes between subsequent cohorts of students within schools after accounting for observable student characteristics (see Appendix B for details). The purpose of the control schools is to adjust for factors (calendar time and cohort) shared by all students in the same grade. The main advantage of DiD is that it accounts for all time-invariant differences between schools, such as stable school, teacher, and student characteristics, irrespective of proxies for these differences. The design may still give biased estimates, however, if there are unobserved differential changes in intervention and control schools that are concurrent with the introduction of the program, but not part of it.

The key assumption is that the evolution of the outcome over time would be parallel for both OBPP schools and control schools in the *absence* of the OBPP intervention (net of the effects of changes in observed student characteristics). If this common trend assumption does not hold, and there are systematic differences in trends between program and control schools, then the effect estimates will be biased. For instance, some regions have a higher share of students exposed to OBPP. Furthermore, for school dropout, for example, mechanisms are related to labor market conditions that may have differential trends in different regions. The common trend assumption is untestable, but we can indirectly evaluate its credibility by comparing program and non-program schools’ trends before implementation. As discussed in Online Appendix B, the common trends assumption seems to hold, as the difference between program and control schools before the implementation (net of differences explained by time-varying covariates and general time trends) is stable.

## Results

### Associations Between School-Level Bullying and Long-Term Outcomes

Because we expect program effects to operate mainly via fewer students involved in bullying, the impact of OBPP will depend on the change in the number of victims (and bullies) multiplied by the long-term consequences of being a victim (or bully). Information on bullying for cohorts exposed to OBPP does not include control schools. However, for more recent cohorts, the Annual National Pupil Surveys of all 7th graders in Norway provide anonymous student-based data describing the proportion of victims and bullies, aggregated to the school level.

Associations between bullying and long-term student outcomes at the school level are not identical to corresponding associations at the individual level. Even if the school-level associations are mainly caused by consequences for victims and bullies, they also capture potential externalities such as improvement in the learning environment for students not directly involved in bullying and adverse effects of observing bullying for bystanders (Rivers et al., 2009). School-level associations may also differ from individual-level associations for other reasons. Because fewer victims typically mean less perpetration (i.e., the correlation between school-level bullying victimization and school-level bullying perpetration of 0.36), the estimated effects of bullying victimization at the school level capture some of the effects of bullying perpetration, and vice versa.

In our sample of schools, the proportion of 7th-grade students who are bullied and bully others are 8.5% and 3.3% in the cohorts 2007–2012. As expected, when looking at bivariate associations, student cohorts in schools that have a greater proportion of 7th graders who bully or are being bullied have lower mean academic performance three years later (at the final exam in 10th grade), a higher proportion of students not completing upper secondary education at age 20, and a higher proportion of students who have committed a crime by the age of 19 (Table 2).

**Table 2:** Association between the share being bullied, the share being perpetrators, and the share being bully-victims in the 7th-grade school-cohort and school-cohort-level outcomes

	Victims		Perpetrators	
	(1)	(2)	(3)	(4)
	Unadjusted	Adjusted	Unadjusted	Adjusted
Examination grades	−0.7231^***^	−0.2283^***^	−0.9979^***^	−0.2973^***^
(Mean = 0.00, SD = 0.32)	(0.0500)	(0.0380)	(0.0708)	(0.0573)
Charged (by 19)	0.1243^***^	0.0650	0.2121^***^	0.1085^*^
(Mean = 0.15, SD = 0.09)	(0.0203)	(0.0352)	(0.0279)	(0.0516)
Up. sec. educ. (by 20)	−0.3167^***^	−0.1011^*^	−0.3494^***^	−0.0860
(Mean = 0.64, SD = 0.13)	(0.0332)	(0.0475)	(0.0454)	(0.0672)
Individual control variables	No	Yes	No	Yes
School fixed effects	No	Yes	No	Yes

Standard errors clustered at the school level in parentheses. The included school cohorts are in grade 7 in the school years 2006/2007 to 2011/2012, and the response rate is above 90%. The number of observations for examination grades is 12,272 and 4502 for charged and upper secondary education completion. Observations are averages at the school-cohort level, and we included analytical weights (number of students in the school cohort) to account for heteroscedasticity due to different school sizes. Bullying victimization is defined as the share of the school cohort during 7th grade that reports being bullied at least 2–3 times a month. Bullying perpetration is defined similarly, as the share of the school cohort that reports bullying others at least 2–3 times a month. In 7th grade, the share being bullied is 8.5%, the share being perpetrators is 3.3%, and the share being bully-victims is 1.1%. Results in columns 1 and 3 are estimated using the regress command, while results in columns 2 and 4 are estimated using the areg command to capture the school fixed effects. All in Stata 16.0

^*^p < 0.05; ^**^p < 0.01; ^***^p < 0.001

Bivariate associations between bullying and long-term individual outcomes are likely confounded (Silberg & Kendler, 2017; Singham et al., 2017), whether bullying is measured at the individual or the school level (as here). To get closer to a causal effect of bullying involvement, we compare subsequent school cohorts within the same school (school fixed effects) after netting out the influence of individual control variables (columns 2 and 4). This reduces the bivariate associations by about 50–70%. While we are reluctant to interpret even these conditional within-school associations as causal effects of school-level bullying, the estimates are informative about the long-term effects of victimization and perpetration at the school level.

Even the likely upwardly biased estimates in Table 2 are fairly tight, suggesting that the potential population-level effects of bullying prevention are quite small. The effects are similar for perpetration and victimization, although the association between victimization and criminal charges is weak as expected. The estimates provide useful input for expected program effects.

### Effects of the OBPP program

Since Table 2 estimates serve as the illustrative maximal population-level outcome effects of decreasing bullying victimization and bullying perpetration by a given percentage point, we can multiply them with the OBPP program effects to benchmark the potential program effect on the long-term outcomes. Based on a bully victimization reduction of 4 percentage points (Olweus & Limber, 2010) and the effect of bully victimization on examination grades being −0.2283, we predict that the program would improve the average examination grade by 0.0091 or close to 1% of a standard deviation.^2^ By the same argument, reducing perpetrators by two percentage points contributes to an examination grade improvement of 0.0059. Thus, the prediction is that the maximal effect of the OBPP would be an increase in the average student grade by 1.5% of a standard deviation (0.0091 + 0.0059 = 0.015). By similar logic, we expect that OBPP at best would reduce criminal charges by 0.5 percentage points and upper secondary dropout by 0.6 percentage points. These estimates show that the program’s potential for reducing *population-level* long-term adversity is minimal. Nevertheless, even such small effects are socially relevant as they add benefits to the primary purpose of the program. School dropout and youth crime are associated with adverse outcomes throughout adult life, and the accumulated costs for even a few individuals can be substantial.

Estimated program effects of OBPP on individual student outcomes using DiD models are displayed in Table 3 (panel I). In some cases, the effect estimates are positive or negative, but they are consistently small and, in no case, statistically significant. We find no indications of program effects on any of the outcomes. The estimates show no clear trend as neither of them increases or decreases with time since implementation. To raise statistical power, the cohort-specific coefficients were combined into an average OBPP effect across cohorts 1 to 4 and across cohorts 2 to 4, presented in panel II. Again, the point estimates are small, and none of the average effects is statistically significant (at 25% significance level or lower).

**Table 3:** Effect estimates and summary post-estimates

	(1)	(2)	(4)
	Examination grades	Complete upper sec. educ. (by 21)	Charged (by 20)
Panel I: Effect estimates			
Years after baseline			
1	−0.0166	−0.0103	0.0007
	(0.0179)	(0.0060)	(0.0060)
2	−0.0004	−0.0035	−0.0004
	(0.0163)	(0.0061)	(0.0063)
3	−0.0229	0.0005	0.0070
	(0.0173)	(0.0059)	(0.0060)
4	−0.0148	−0.0025	−0.0015
	(0.0185)	(0.0063)	(0.0061)
Panel II: Summary post estimates			
After 1–4 years	−0.0137	−0.0039	0.0015
	(0.0122)	(0.0040)	(0.0045)
After 2–4 years	−0.0127	−0.0018	0.0017
	(0.0129)	(0.0044)	(0.0048)
Individual control variables	Yes	Yes	Yes
School fixed effects	Yes	Yes	Yes
N	521,870	527,002	487,891

Standard errors clustered at the school level in parentheses. The coefficients are estimated using the xtreg command in Stata 16.0. The outcome metric is z-standardized for examination grades and the observed share for criminal charges and upper secondary education completion. Coefficients in panel I are shown graphically in Appendix Figure C2

^*^p < 0.05; ^**^p < 0.01; ^***^p < 0.001

## Effects by Fidelity of Implementation

Fidelity to the OBPP program’s intent and implementation of core program components are essential to achieve a reduction in bullying. Our main effect estimates, which reflect the program’s average effects as implemented, may accordingly underestimate the program’s maximal potential. Unfortunately, no fidelity data was collected during the implementation stage for the OBPP schools included in this study. However, we observed whether schools continued to use the Olweus Bullying Questionnaire (OBQ) several years after the first implementation (Olweus et al., 2020). The use of this questionnaire was used as a proxy for the fidelity of implementation. Before implementing OBPP, schools that continued to use OBQ differed from other OBPP schools and control schools as they had students who were found later to have higher examination grades and better completion of upper secondary education. Most of these differences are explained by observed student characteristics (Appendix Table C2). Long-term intervention effects are similar for OBPP schools that continue using the program and other OBPP schools (Appendix Fig. C11).

### Robustness Checks: Regional Time Trends and Matched DiD (M-DiD)

Two additional robustness checks are performed to check whether the results are sensitive to the common trends assumption. First, we have included school county-specific cohort fixed effects and municipality-specific cohort fixed effects to allow for differential regional trends. In these more flexible models, identification is based on differences in the three outcomes across students of different cohorts within schools, adjusted for the average differential between cohorts in that region. School county and school municipality have, on average, about 2250 and 100 students for each fixed effects group, respectively. With region specific cohort fixed effects, the results are slightly less precise, but the effect estimates are of similar magnitude and direction as in the main model specification (Appendix Fig. C4).

Second, we checked the robustness using a matched DiD model. The main concern in our DiD model is that schools that implement OBPP are on a different development trajectory than the typical control school. To identify program effects in the DiD setup, we need a group of comparison schools that serve as a counterfactual, i.e., how OBPP school students would have performed if the OBPP had not been implemented. While the main model specifications use all untreated schools as control schools, another option is to use a subset of matched control schools only (Cook et al., 2008; but see Daw & Hatfield, 2018). We find similar results by matching on pre-intervention levels in the outcome variables as in the main model specifications (see Online Appendix D for details and results).

## Discussion

By reducing bullying and victimization behavior, it has been suggested that anti-bullying programs *could* have favorable side effects in terms of better long-term student outcomes. However, the empirical evidence supporting claims that programs may, in the long term, improve academic outcomes and lower anti-social behavior that may cluster with or follow bullying behavior (e.g., youth crime) is lacking. This paper empirically examined the longer-term effects of the OBPP bullying prevention program in Norway on academic achievement, school dropout, and criminal charges. While the OBPP program is widely acknowledged internationally as one of the most successful bullying prevention programs (Ttofi & Farrington, 2011), no statistically significant effects of OBPP were found on any outcome variables.

The insights to be drawn from non-significant results hinge crucially on the precision of the estimates. If the estimates are “imprecisely zero”, we cannot reject even large effects, and not much has been learned about the possible effects of the program. One way of approaching the estimates’ statistical precision is to check whether plausible effect estimates are within the 95% confidence intervals. Our benchmarked potential program effects from the school-level associations between bullying and long-term outcomes (Table 2) and OBPP effects on bullying discussed above are reasonable predictions of realistic results. If these potential program effects fall outside the estimated 95% confidence intervals, the evidence suggests smaller (larger) program effects than expected. Conversely, if the confidence intervals include these potential effects, we cannot reject either zero or realistic program effects, and not much has been learned.

For examination grades in grade 10, the estimated confidence interval ranges from − 0.0376 to 0.0102 (panel II in Table 3, after 2–4 years). Adjusting the confidence intervals for misclassification because of the linking of students to schools widens these ranges slightly, from − 0.0418 to 0.0113. This result suggests that the OBPP effect on examination grades is likely to be lower than the suggested maximal effect of 1.5% of an SD (Table 2). The upper end of the estimated confidence intervals of the program effects instead indicates that, at best, OBPP improves examination grades by 1.1% of an SD for the average student exposed to the program. This result means that an improvement of examination grades of 1.1% or less is consistent with our data, while effects stronger than 1.1% are not.

For completion of upper secondary education, the confidence intervals adjusted for misclassification range from a reduction of 1.3 percentage points to an improvement of 0.44 percentage points, which again indicates that the maximal effect of 0.6 percentage points is unlikely and that the program effects of OBPP are lower than this optimistic maximal effect estimate. However, we cannot reject the intervention effects of about 0.4 percentage points or smaller. For criminal charges, the power to detect realistic intervention effects are even more restricted; the confidence interval adjusted for misclassification ranges from − 0.0081 to 0.0115 and contains the potential best-case program effects of − 0.005.

Overall, the confidence intervals inform the scope of program effects on long-term outcomes. Based on the 95% confidence intervals (adjusted for misclassification), we conclude it is unlikely that OBPP improves examination grades by more than 1.1% of an SD, increases upper secondary completion by more than 0.4 percentage points, and reduces criminal charges by more than 0.8 percentage points. The limited ability to reject a range of plausible intervention effects follows from the fact that potential effects are small and that having the precision to identify such small effects is hard, even with large-scale register data.

This paper adds to a small literature suggesting that expectations of finding long-term effects of early universal prevention programs *at the population level* should be low (Averdijk et al., 2016). Importantly, the lack of long-term effects in this study has little to do with the program’s effects on bullying problems. When studied at the school or population level, a low initial prevalence of the intervention’s target problem clearly limits the potential for long-term effects. The potential population-level gains of bullying prevention efforts depend crucially on both the causal effects of bullying victimization and bullying perpetration on the long-term outcomes *and* the reduction in the number of victims and bullies due to the program. Concurrent with previous research on the individual level, we find that the proportion of bullies and victims at the school cohort level is associated with a higher mean risk of later life adversity. However, most students are not involved in bullying, and they are, therefore, largely unaffected by the program. This point is important because the long-term program effects on the average student (or school cohort) likely operate mainly through a reduction in the proportion of students who are victimized or are bullies.

There are other potential explanations for the lack of long-term effects. Even eliminating bullying may result in only minor improvements in academic failure and youth crime if the causal effects of bullying on these outcomes are small (Schoeler et al., 2018; Silberg & Kendler, 2017). For example, the potential influence of bullying perpetration on later anti-social behavior is not well understood (Ttofi et al., 2012), and bullying others may not be an independent cause of later youth crime. Although we find an association between bullying perpetration and later youth crime in this study, we cannot rule out confounding as an explanation for these school-level associations.

Moreover, most of the program-exposed students in the current study (grades 4–7) spend their remaining years of schooling in schools without a systematic, effective intervention against bullying. It is reasonable to assume that some students escaping victimization and perpetration due to the intervention are likely to be the victims and bullies when entering a new school without such programs (e.g., Connolly & Beaver, 2016). This negative exposure may counteract a positive development and limit the possible effects of the earlier intervention at the elementary school for a sizeable proportion of the program students. Our study is not able to describe what would be the effects of prolonged bullying prevention efforts throughout the entire school career.

Further, it is reasonable to expect a decay of program effects over the 8-year period from early intervention to an outcome in young adulthood (Schoeler et al., 2018; Ttofi et al., 2011). For example, even if victimization has severe effects on internalizing problems, the long-term effects of internalizing problems on academic and behavioral outcomes are likely to be minor if the health care or family environment offers adequate help to deal with those problems. More generally, the effects of a childhood prevention program on long-term outcomes depend on society’s ability to deal with the consequences of the short-term outcomes the program is intended to avoid.

Despite no long-term effects at the overall population level in this study, there might be important effects for victims and bullies. In the current project, the bullying survey data were linked to schools only and not to register data for individual students. The power to detect long-term effects would be stronger with a design permitting mediating effects through student-linked bullying data (Pituch & Stapleton, 2012). With such student-linked data, one promising approach would be to investigate program effects for students who were victims or bullies before the implementation of the OBPP program. With the data available in this project, however, it is not possible to identify program effects for students who escaped bullying due to the intervention, and the effect analyses at follow-up are based on the number of students who had a positive development compared to control schools. Hence, we have a limited opportunity to study the long-term effects of bullying prevention for potential victims and bullies in this study and are largely restricted to study average population-level program effects.

Although our findings point to a limited scope of anti-bullying programs to prevent long-term population-level adversity, they do not speak to bullying prevention efforts’ main objective. Bullying is a form of childhood abuse that has serious implications for the wellbeing of children regardless of any long-term effects, and prevention efforts in schools are accordingly needed to safeguard the human rights of children and improve their wellbeing (Arseneault, 2018; Olweus, 1993; Olweus & Breivik, 2014). Additionally, because of the substantial economic and social burden of academic failure and criminal charges, even small population-level intervention effects may be cost-beneficial and worthwhile from an individual as well as a public health and policy perspective (Cohen & Piquero, 2009; Levin & Belfield, 2007).

## Supplementary Information

Below is the link to the electronic supplementary material.

Supplementary file1 (DOCX 1388 KB)

## Footnotes

1 As a result, we exclude 3147 students who immigrate between school-starting age and program implementation. Including these immigrants does not alter the main results (not shown).

2 Population program effect = Effect of bully victimization * Effect of OBPP on the fraction of bully victims = $-0.2283*-0.04=0.0091$. See Fig. 1 for the rationale behind this calculation.
