# Supplementary material for: The Potential of Anti-Bullying Efforts to Prevent Academic Failure and Youth Crime. A Case Using the Olweus Bullying Prevention Program (OBPP)
Source: Prev Sci. 2021 May 16;22(8):1147–58. doi: 10.1007/s11121-021-01254-3 (PMC8541967; doi:10.1007/s11121-021-01254-3)
Supplement: Supplementary file 1 — Supplementary file1 (DOCX 1388 KB) [file 11121_2021_1254_MOESM1_ESM.docx]

Supplementary Online Appendix for

**The potential of anti-bullying efforts to prevent academic failure and youth crime. A case using the Olweus Bullying Prevention Program (OBPP)**

Nicolai Topstad Borgen^1,2^, Dan Olweus^3†^, Lars Johannessen Kirkebøen^4^, Kyrre Breivik^5^, Mona Elin Solberg^5^, Ivar Frønes^1,6^, Donna Cross^7^, and Oddbjørn Raaum^8^

^1^ Department of Sociology and Human Geography, University of Oslo, Norway

^2^ Department of Special Needs Education, University of Oslo, Norway

^3^ Department of Health Promotion and Development, University of Bergen, Norway

^4^ Statistics Norway, Norway

^5^ Regional Centre for Child and Youth Mental Health and Child Welfare, NORCE, Norway

^6^ Norwegian Center for Child Behavioral Development, Norway

^7^ University of Western Australia – Telethon Kids Institute, Australia

^8^ Ragnar Frisch Centre for Economic Research, Norway

^†^ Deceased 20 September 2020.

**Content of supplementary online appendix:**

**Online Appendix A:** School and student identification

**Online Appendix B:** Analytical approach to study long-term effects of OBPP

**Online Appendix C:** Supplementary tables and figures

**Online Appendix D:** Matched DiD

**Online Appendix References**

# Online Appendix A: School and student identification

A national register of compulsory school students does not exist in Norway. To identify the students who attended OBPP schools, we imputed school attendance from residential addresses using links constructed from test score data from later years. We use the fact that Norwegian primary school students overwhelmingly attend their local schools (less than 5 percent attend private schools) and impute school assignments based on the residential address.

Register data identifies the residential location of students, in particular the basic statistical units (basic districts). There are about 14,000 such units, which according to Statistics Norway constitute “… small, stable geographical units which may form a flexible basis to work with and present regional statistics. (...) geographically coherent areas. (...) homogeneous, with respect to nature and basis for economic activities, conditions for communications, and structure of buildings.” The units have 0-6000 inhabitants (mean = 379).

From 2007 and onwards, students take standardized tests in grades 5 and 8 (later also in grade 9). Results are recorded at the student level and include school id and student id. Based on the standardized test data for 5^th^ graders in 2007-2009, we found the most frequently attended school for students in each basic statistical unit. We then imputed school characteristics, including OBPP participation, using these modal schools.

Imputing school attended from residential address will cause some misclassification of schools attended, and thus of program exposure: Some students classified as attending an OBPP school where the program was implemented will not have attended this school, while some classified as not exposed will have attended.^[[1]](#footnote-1)^ By making two key assumptions, however, we can assess the implications of attenuation bias for our results.

The first assumption is about the type of misclassification. Some schools or groups of students will tend to have more misclassification than others do (e.g., due to the geography and size of catchment area), and they may also differ in other ways. For example, students in cities may have more available school options, including private schools, compared to those in rural areas. These students may have both a different share of misclassification and different characteristics and outcomes than other students. However, since our research design accounts for unobserved persistent differences in the outcomes of students in different schools as well as family background for each student, it is unlikely that misclassification is correlated with unobserved factors affecting outcomes.

In essence, we assume that misclassification is conditionally random, which will attenuate the effect estimate (e.g., Lewbel 2007). If a share *a* of students classified as OBPP were correctly classified (“true positive”) and the share *b* among those classified as not OBPP were in fact exposed to OBPP (“false negative”), the difference in true program exposure between students classified as OBPP or those without OBPP would be *a-b*<1. This will bias our effect estimates towards zero. However, knowing *a* and *b* we can inflate our effect estimates with a factor 1/(*a-b*) to get an assumedly unbiased estimate of the true effect.

Our second problem is that we can only assess misclassification for more recent student cohorts. Therefore, the second key assumption is that we can extrapolate the misclassification from these samples to older cohorts of students. We observe the schools where students took national standardized tests in grade five and their home neighborhood from 2007 onwards (born 1997 and later). This allows estimation of *a* and *b* for the recent student cohorts. Our analyses indicate a school prediction based on neighborhood gives *a*≈0.9 and *b*≈0.0 in years close to the year the standardized tests are taken (Appendix Figure A1). Thus, despite some misclassification, we expect 85-90% of those classified as exposed to OBPP are indeed exposed (the remaining 10-15% attend a control school). Further, very few of those classified as not exposed are exposed, reflecting that a minority of schools are program schools. Thus, students incorrectly assigned to a given control school will mostly attend some other control school and therefore get the correct treatment status.


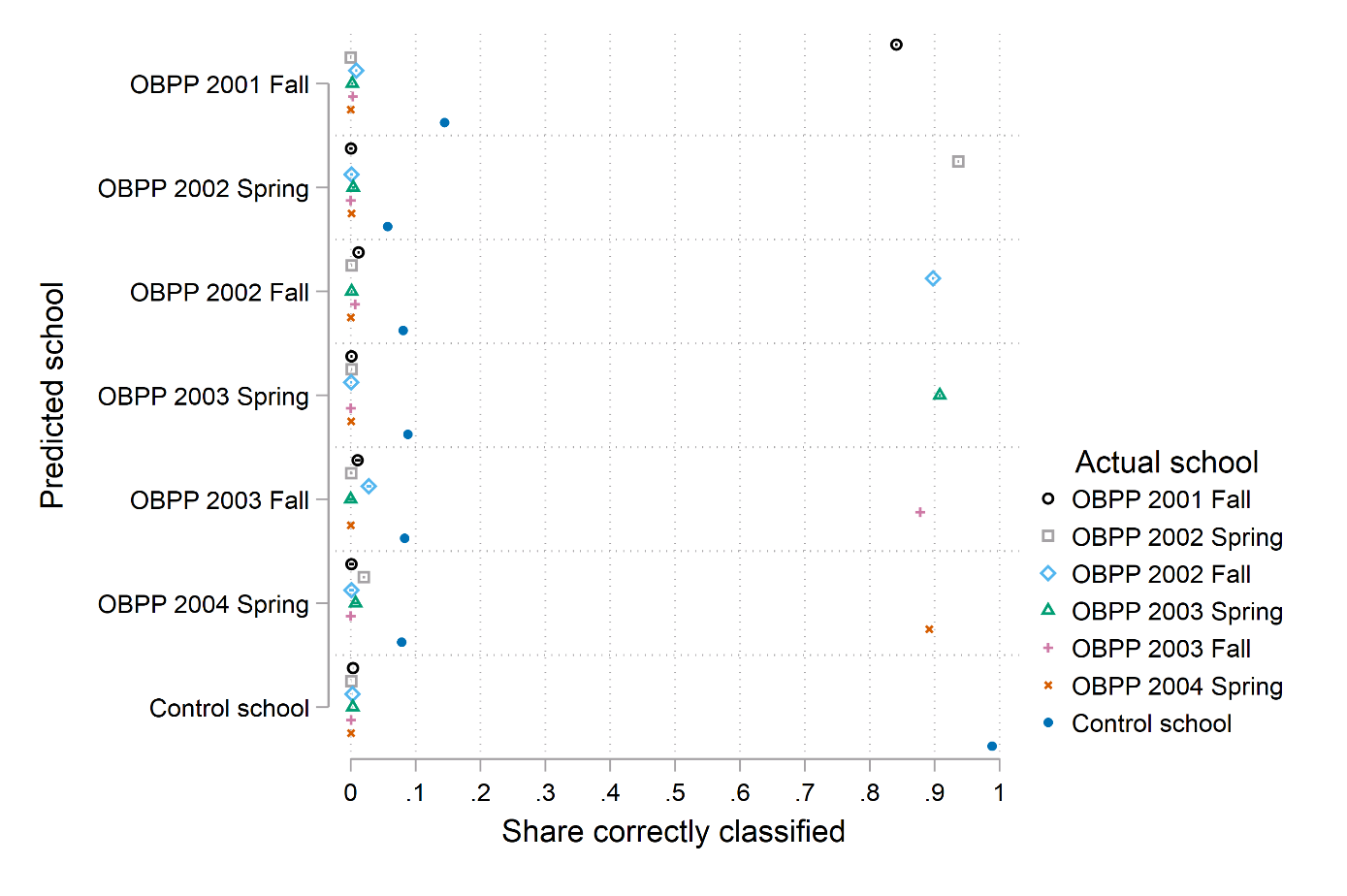


**Appendix Figure A1:** Share of students correctly classified as OBPP schools and control schools based on a predicted treatment from the residential address.

Note: Actual school is observed for cohorts completing standardized national tests in 5^th^ grade between 2007 and 2012.

We are mainly concerned about school attendance around the time of the introduction of the OBPP program, which is earlier than the years used to impute schools (2001-2004 vs. 2007-2009). Since we have data on the elementary school from 2007 and onwards (through test score data), we cannot test the predicted school approach's precision for the birth cohorts used in the effect estimation. However, we have no evidence of large school assignment changes in the relevant period, and private school attendance is stable and very low.

Further, data on student counts for each school exist for all years from 1992 and onwards, allowing us to compare our predicted school counts with schools' registered student numbers. A large majority of schools have existed since the start of the school register in 1992 (the schools present both in 1992 and 2007 have 96 percent of the students in 2007). Concerning the student count data, these do not exactly correspond to the test score data. While both standardized tests and student counts are performed in the fall, the timing may differ slightly. More importantly, some students are exempted from the standardized tests, and whether and how these are reported varies. Still, we expect our predicted number of students to be highly correlated with the count data.


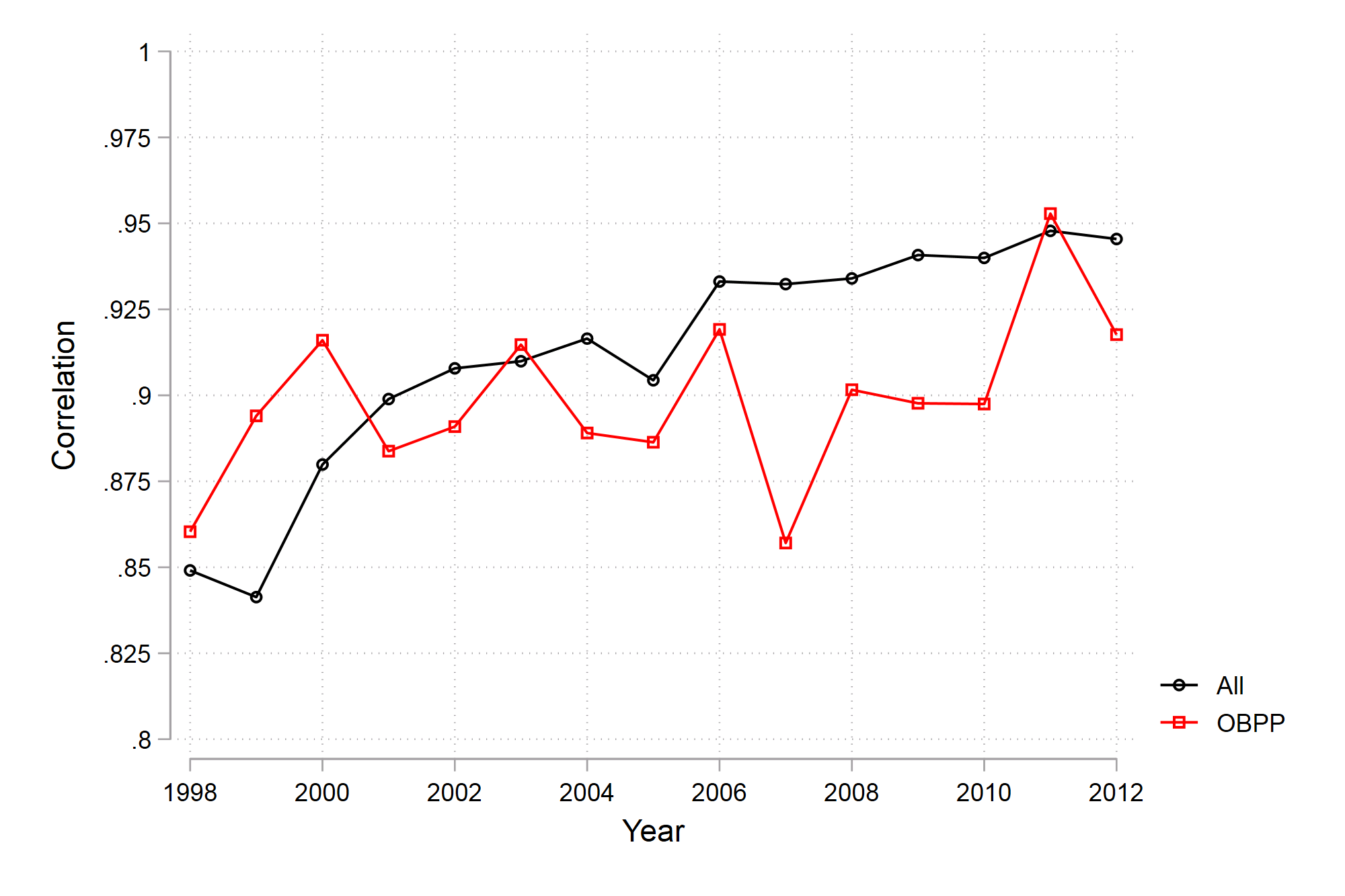


**Appendix Figure A2:** Correlations between the predicted number of students (based on residential address) and student counts from administrative data.

By correlating predicted student numbers with student counts from administrative data, we find consistently high correlations (≈.90). Although the correlations decline slightly with increasing time difference between the cohorts studied and those used for constructing linkages, the correlation is still above .85 at the extremes of our sample (Appendix Figure A2). While it is hard to draw conclusions about *a* and *b* from these correlations without investigating the school attended by individual students, this consistently high correlation gives no reason to believe the correct classification decreases dramatically from *a-b*=0.9 in the years used to generate the address-school links.

Thus, we assume the effect estimates are attenuated by a factor of about 0.9 and can inflate coefficients and standard errors by about 11% (1/0.9=1.11) to adjust for the bias. This adjustment is relatively minor. Additionally, it is based on the subset of the sample for whom we observe standardized tests, and accordingly has some estimation uncertainty. Therefore, we will not explicitly implement this adjustment as part of our estimator but instead, refer to it in our discussion of the results.

# Online Appendix B: Analytic approach to study long-term effects of OBPP

Schools implementing a program like OBPP may differ from other schools (e.g., higher levels of bullying), and we cannot directly compare students from program and control schools. Our strategy is to compare outcomes of different birth cohorts *within the same school* and use a difference-in-difference (DiD) model that accounts for selection into treatment as well as time/cohort effects common to all schools. The DiD model compares outcomes between subsequent cohorts of students within schools after accounting for observable student characteristics. The purpose of the control schools is to adjust for factors (calendar time and cohort) that are shared by all students in the same grade. The main advantage of DiD is that it accounts for all time-invariant differences between schools, such as stable school, teacher, and student characteristics, irrespective of proxies for these differences (Angrist and Pischke 2009).

The unit of observation is the student and the basic model is:

(1) $Y_{ics}=\beta_{0}+\beta T_{cs}+\delta X_{ics}+\gamma_{c}+\mu_{s}+\varepsilon_{ics}$

$Y_{cs}$ is the outcome (e.g., end-of-compulsory school grades or completing upper secondary) of student $i$ belonging to cohort *c* that attended school $s$. $\beta_{0}$ is a constant term, $T_{cs}$ indicates whether a given cohort in a given school was enrolled after the implementation of OBPP ($T_{cs}=1$), $\gamma_{c}$ is dummy variables for birth cohort (i.e., birth cohort fixed effect), $\mu_{s}$ is dummy variables for schools (i.e., school fixed effect), and $X_{ics}$ are observed student characteristics variables (female, fathers’ and mothers’ education and earnings, immigrant background). Cohort refers to the year a student exits primary school (and exposure to OBPP ends). $\varepsilon_{ics}$ is a residual, capturing unexplained variation in results at the student level.

The key identifying assumption is that the evolution of the outcome over time would be parallel for both OBPP schools and control schools in the *absence* of the OBPP intervention (net of the effects of changes in observed student characteristics). If this common trend assumption does not hold, and there are systematic differences in trends between program and control schools, then the effect estimates will be biased. For instance, some regions have a higher share of students exposed to OBPP. Furthermore, for school dropout, for example, mechanisms are related to labor market conditions that may have differential trends in different regions.

The common trend assumption is untestable, but we can evaluate its credibility indirectly by comparing trends for program and non-program schools before implementation. If the assumption of common trends holds, we should expect a stable difference between the program and the control schools *before* the implementation (net of differences explained by time-varying covariates and general time trends). During the pre-program years, we expect no (significant) differences between cohorts within the same school. In Appendix Figure C2, we estimate the OBPP coefficient before and after implementation:

(2) $Y_{ics}=\beta_{0}+\sum_{p=-4}^{4} \beta_{p}T_{csp}+\delta X_{ics}+ \gamma_{c}+\mu_{s}+\varepsilon_{ics}$

, where $\beta_{p}$ parameters identify any pre-program differentials (p<0) and post-implementation effects (p>0) as $T_{csp}=1$ when the outcome of the cohort is measured *with a time distance of p years* since the implementation of the program. $\beta_{-1}$ is set equal to 0 and provides a reference for the effect estimates for the other years. For example; $\beta_{3}$ measures the effect on $Y$ for students exiting primary school three years after the implementation of OBPP relative to the outcomes of students exiting primary school just before implementation, i.e., the effect of being exposed to OBPP for three years (grades 5-7). The credibility of the common trend assumption is tested by examining if there are no differences between cohorts within the same school before the implementation, i.e., $\beta_{p}=0$ for p<0 (net of differences explained by time-varying covariates and general time trends).

The results in Appendix Figure C3 suggest the assumption of common trends holds, and consequently that the effect estimates are valid. Additionally, the results are similar when allowing time trends to differ by region (Appendix Figure C4), and the results are similar when using a matching DiD model (Appendix Figure D2). Consequently, in the main analyses, we opt for a more stripped-down DiD model where we estimate the effects of OBPP in years since the implementation of the program relative to the (average of the) pre-intervention years:

(3) $Y_{ics}=\beta_{0}+\sum_{p=1}^{4} \beta_{p}T_{csp}+\delta X_{ics}+ \gamma_{c}+\mu_{s}+\varepsilon_{ics}$

Linear regression analysis is used with both continuous outcomes (examination grades) and binary outcomes (charged and completion of upper secondary education). For the binary outcomes, ordinary least squares is preferred over logistic regression or probit because of the problem of unobserved heterogeneity, and to ease interpretation of the estimates (Mood 2010); however, results using unconditional fixed effects logistic regression is similar to the main finding (Appendix Figure C12). Students $i$ are nested within cohort *c* within schools $s$ and there is a concern that unobserved characteristics or shocks make the residuals $\varepsilon$ correlated within schools. We cluster standard errors at the unit of the fixed effect (overall school level) in all analyses to adjust for these correlations (Cameron and Miller 2015). ^[[2]](#footnote-2)^

# Online Appendix C: Supplementary tables and figures

**Appendix Table C1:** Number of schools that have implemented OBPP by the baseline of implementation. Program effects by baseline semester is shown in Appendix Figure C5.

|  | Included OBPP schools | Excluded OBPP schools |
| --- | --- | --- |
|  |  |  |
| 2001 Fall | 51 | 1 |
| 2002 Spring | 43 | 0 |
| 2002 Fall | 54 | 1 |
| 2003 Spring | 109 | 7 |
| 2003 Fall | 9 | 4 |
| 2004 Spring | 34 | 4 |
|  |  |  |
| Total | 208 | 17 |

**Appendix Table C2:** Pre-intervention differences between 70 schools that continue using OBPP (A-schools), 102 schools that do not (B-schools), and 157 schools we do not know (Missing), compared to control schools.

|  | (1) | (2) | (3) | (4) | (5) | (6) |
| --- | --- | --- | --- | --- | --- | --- |
|  | A-schools | B-schools | Missing | A-schools | B-schools | Missing |
|  |  |  |  |  |  |  |
| **Panel I: Outcome variables** | | | |  | | |
| Completed up sec. educ (by 21) | 0.0374^**^ | -0.0043 | -0.0079 | 0.0142 | -0.0078 | -0.0060 |
|  | (0.0130) | (0.0090) | (0.0177) | (0.0080) | (0.0068) | (0.0127) |
| Examination grades | 0.0957^*^ | 0.0205 | -0.0047 | 0.0260 | 0.0091 | 0.0002 |
|  | (0.0375) | (0.0262) | (0.0369) | (0.0172) | (0.0179) | (0.0280) |
| Charged (by 20) | -0.0109 | 0.0008 | -0.0004 | -0.0027 | 0.0022 | -0.0023 |
|  | (0.0078) | (0.0066) | (0.0133) | (0.0067) | (0.0059) | (0.0109) |
|  |  |  |  |  |  |  |
|  | | | |  | | |
| Parents' education | 0.2191^*^ | 0.0535 | -0.0146 |  |  |  |
|  | (0.0950) | (0.0565) | (0.0893) |  |  |  |
| Parents' earnings (in 1000 NOK) | 30.4033^**^ | 1.3042 | -5.5233 |  |  |  |
|  | (10.9503) | (6.9339) | (12.7047) |  |  |  |
| Immigrant background | 0.0286^*^ | -0.0053 | 0.0402 |  |  |  |
|  | (0.0123) | (0.0040) | (0.0306) |  |  |  |
|  |  |  |  |  |  |  |
| Control variables | No | No | No | Yes | Yes | Yes |

Note: Only pre-intervention cohorts are included (born 1986-1988). Standard errors clustered at the school level in parentheses.

^*^ *p* < 0.05, ^**^ *p* < 0.01, ^***^ *p* < 0.001

**Appendix Figure C1:** Share of students in various birth cohorts exposed to OBPP between 1 and 4 years.

Note: Students exposed five or more years are included as not exposed in this graph but are excluded from the effect estimates.


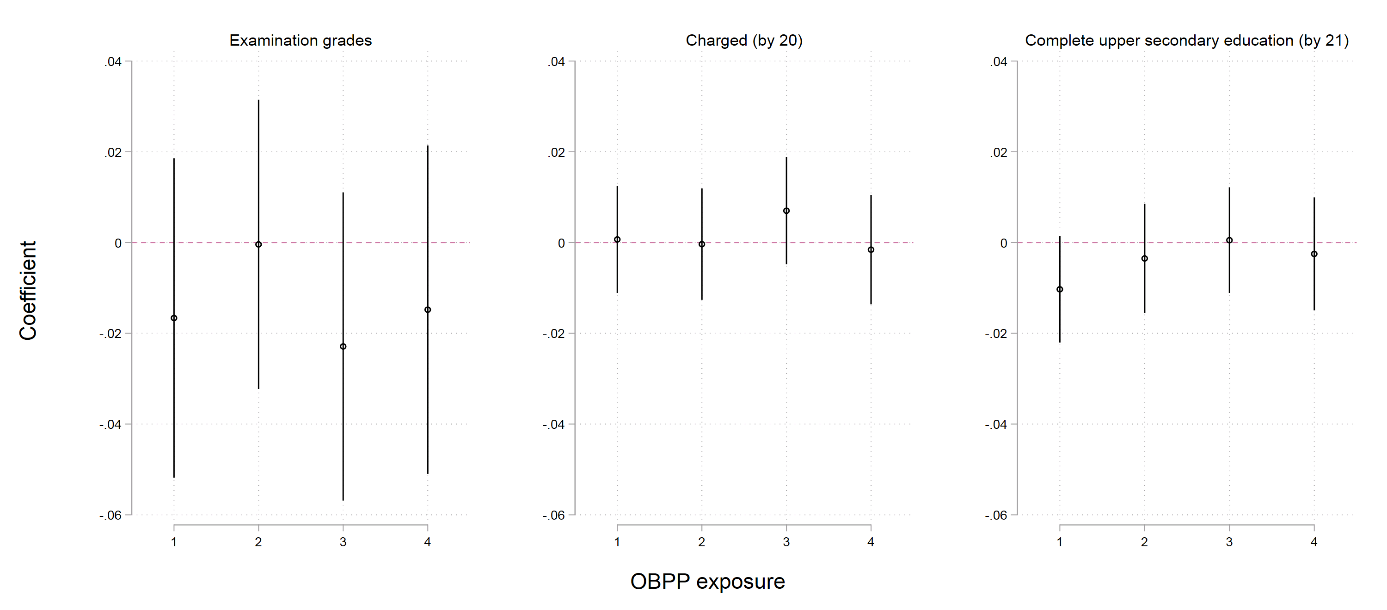


**Appendix Figure C2**

*Effect Estimates With 95% CI.*

Note: Outcome metrics: Standardized for examination grades. Observed share for criminal charges and completion of upper secondary education.


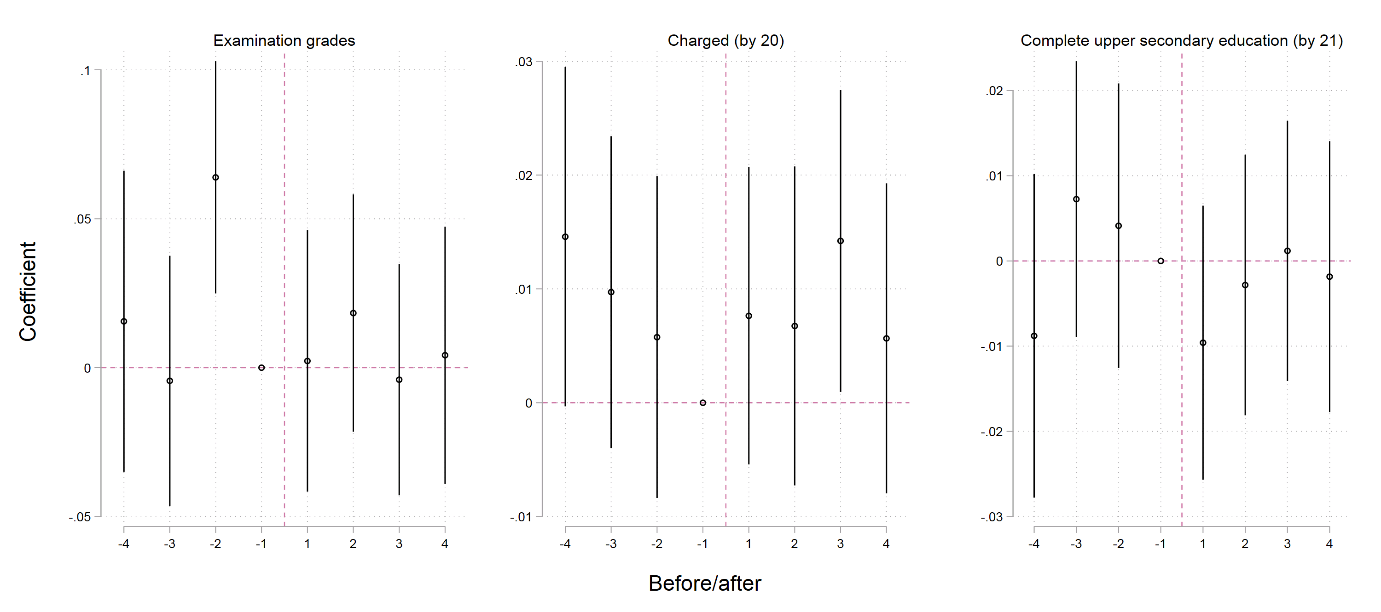


**Appendix Figure C3:** Effect estimates (after) and pre-program heterogeneity with 95% CI.

Note: Outcome metrics: Standardized for examination grades. Observed share for criminal charges and completion of upper secondary education.


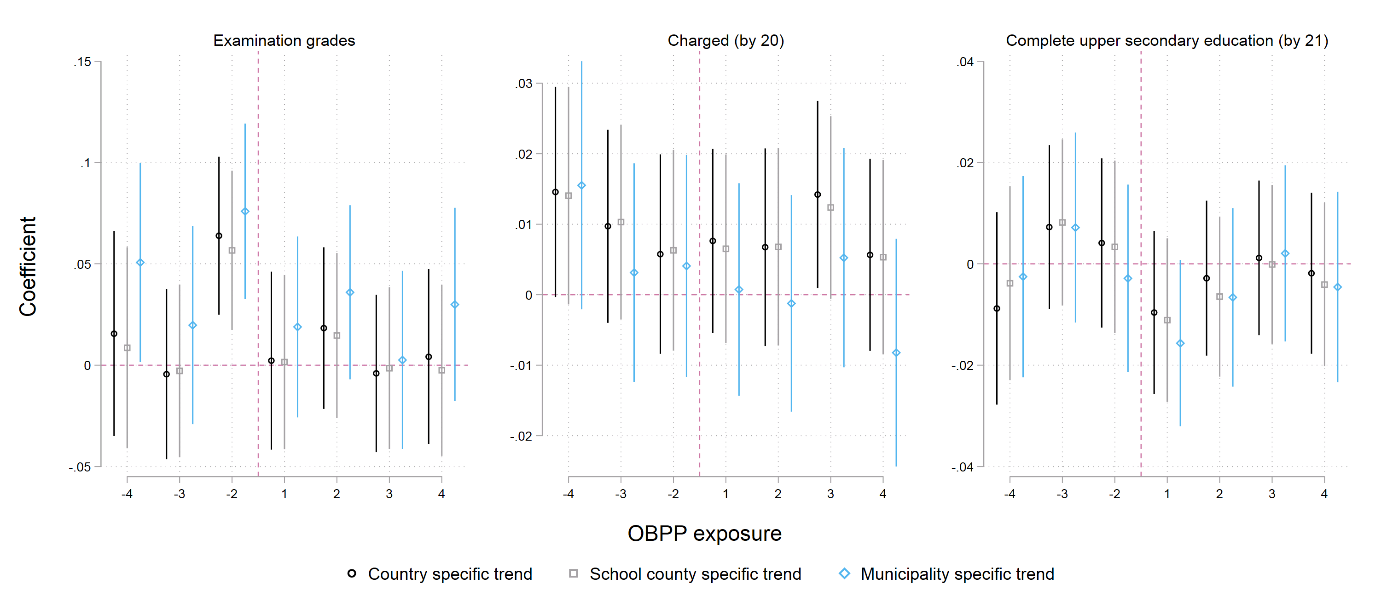


**Appendix Figure C4:** Effect estimates (after) and pre-program heterogeneity with region-specific trends with 95% CI.

Note: Outcome metrics: Standardized for examination grades. Observed share for criminal charges and completion of upper secondary education. Results with school county and municipality specific trends include interaction variables between the year of birth and school county and municipality, respectively.


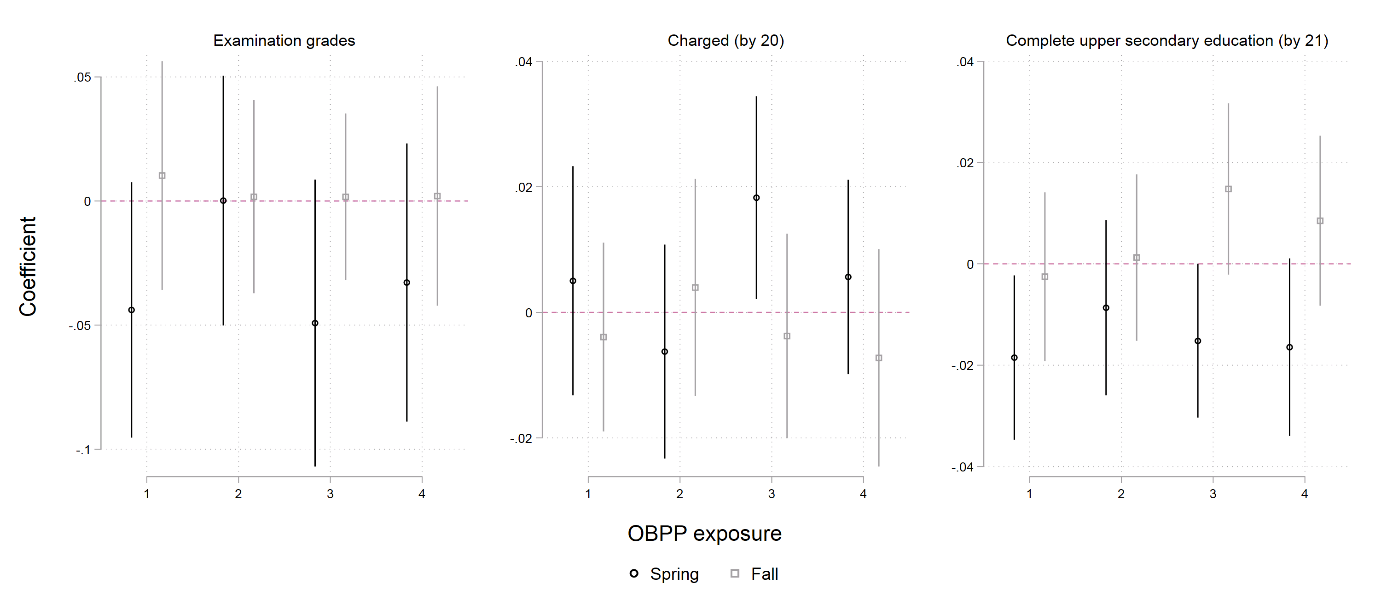


**Appendix Figure C5:** Effect estimates by baseline semester with 95% CI.

Note: Outcome metrics: Standardized for examination grades. Observed share for criminal charges and completion of upper secondary education.


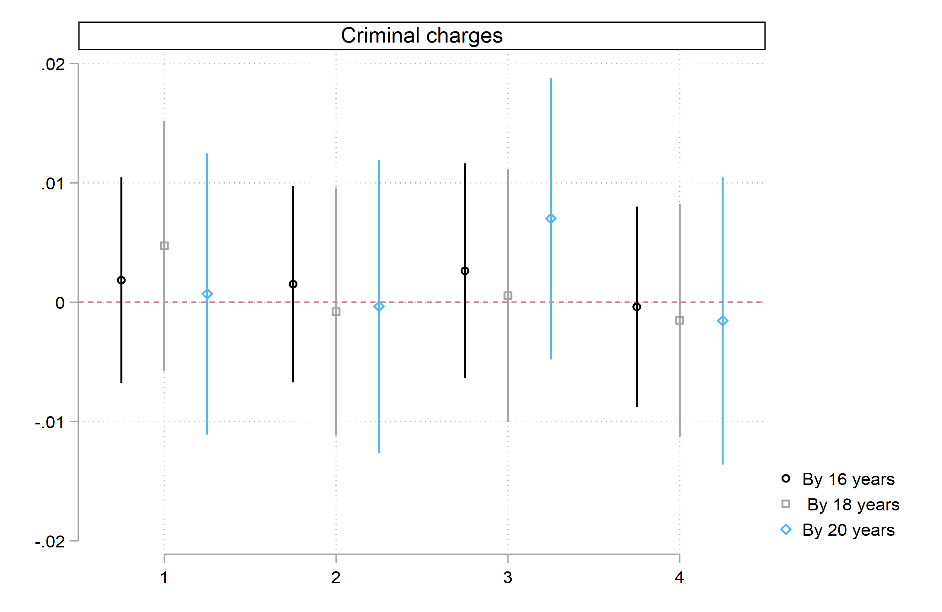


**Appendix Figure C6:** Effect estimates on criminal charges by age with 95% CI.

Note: Outcome metrics: Observed share for criminal charges.


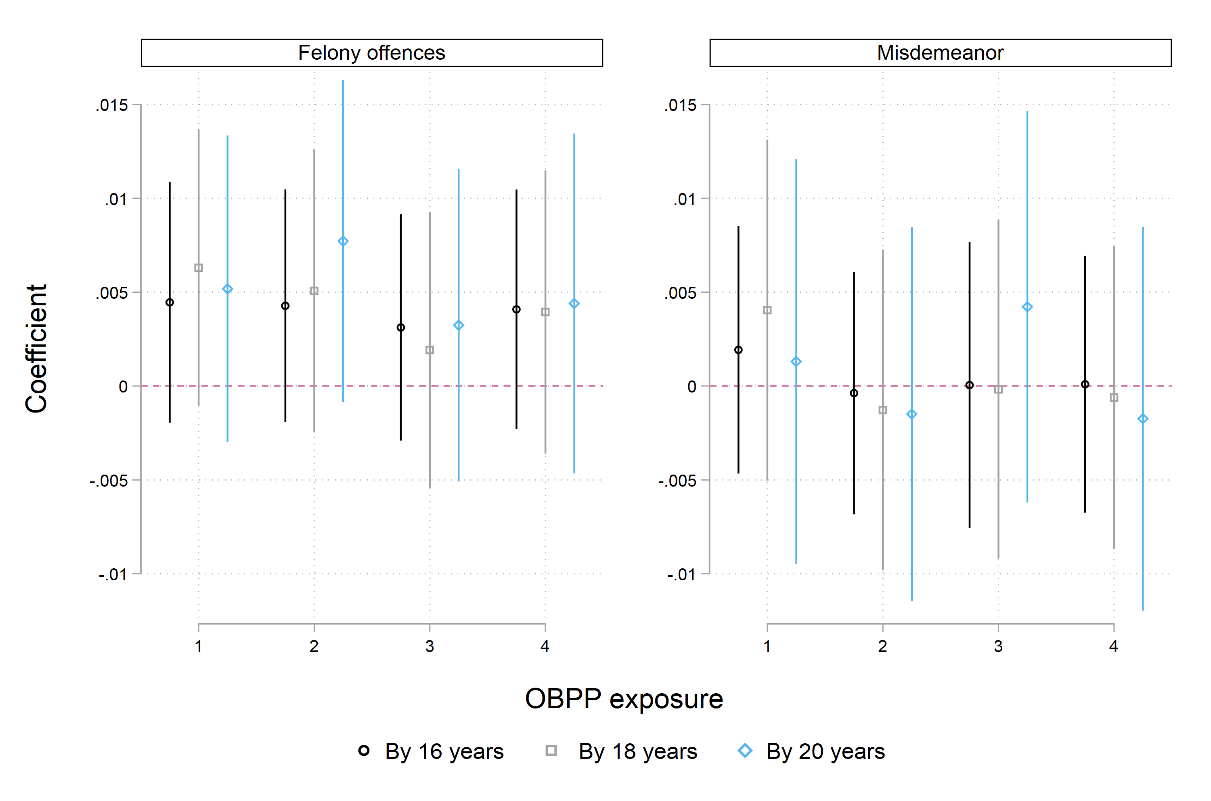


**Appendix Figure C7:** Effect estimates by type of criminal charge and age with 95% CI.

Note: Outcome metrics: Observed share for criminal charges.


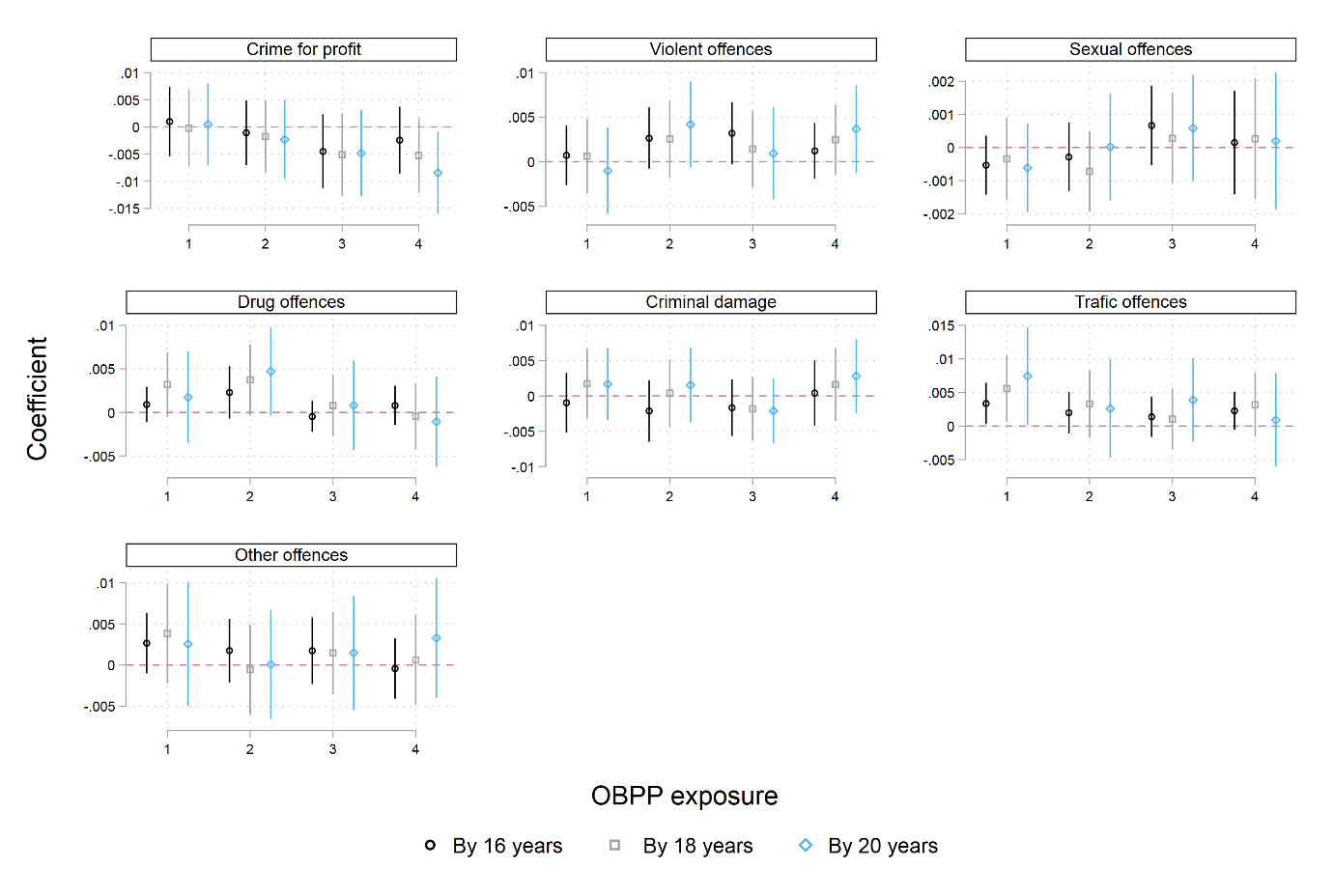


**Appendix Figure C8:** Effect estimates by detailed type of criminal charge and age with 95% CI.

Note: Outcome metrics: Observed share for criminal charges.


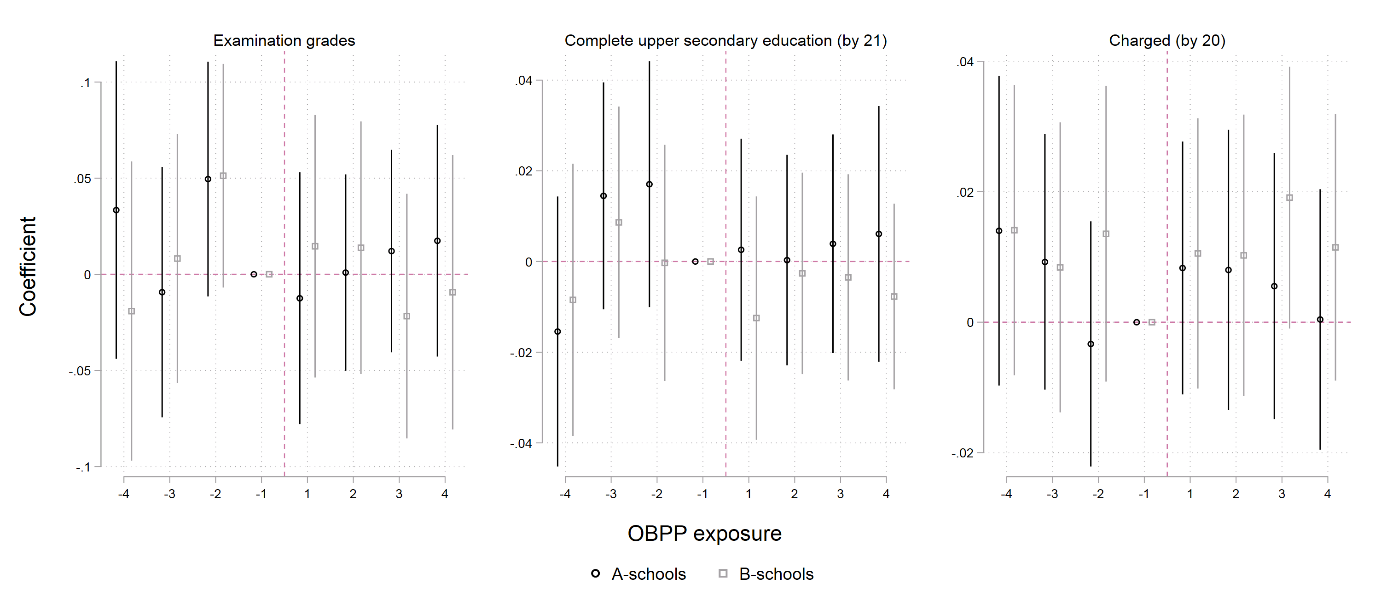


**Appendix Figure C9:** Effect estimates (after) and pre-program heterogeneity by A- and B-schools with 95% CI.

Note: Outcome metrics: Standardized for examination grades. Observed share for criminal charges and completion of upper secondary education.


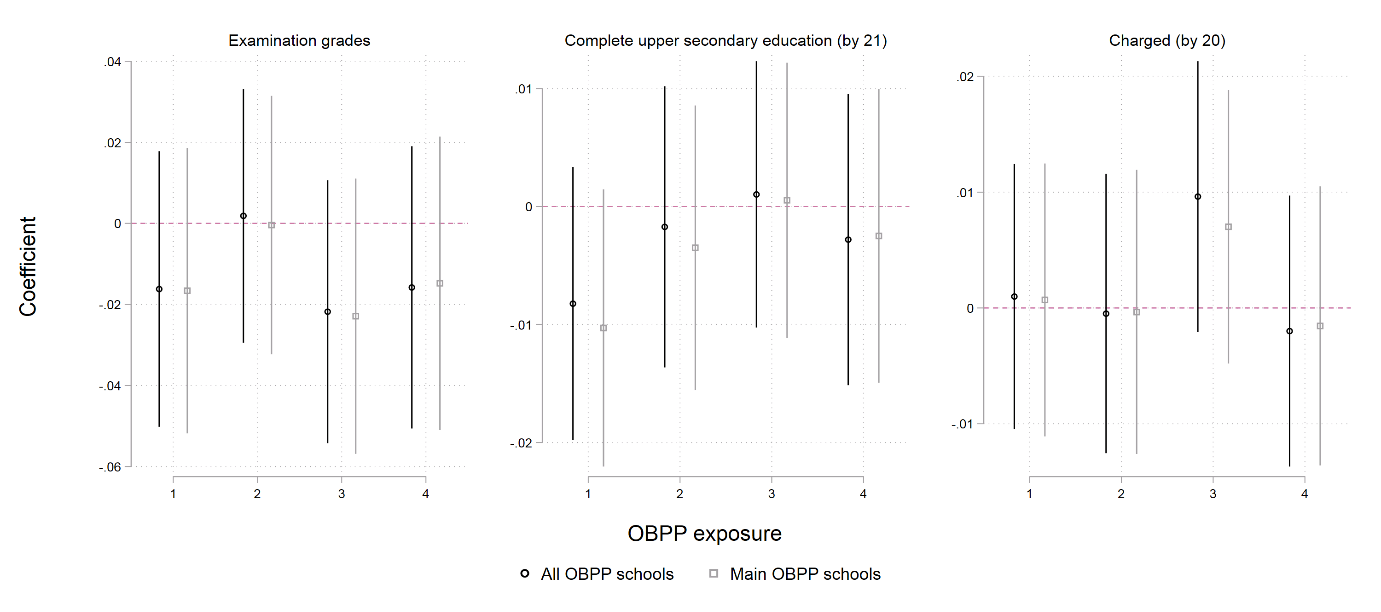


**Appendix Figure C10:** Effect estimates with all OBPP elementary schools compared to the main OBPP schools.

Note: The results for the main OBPP schools exclude 17 of the 225 elementary schools because they had problems with implementing the program during the implementation period.


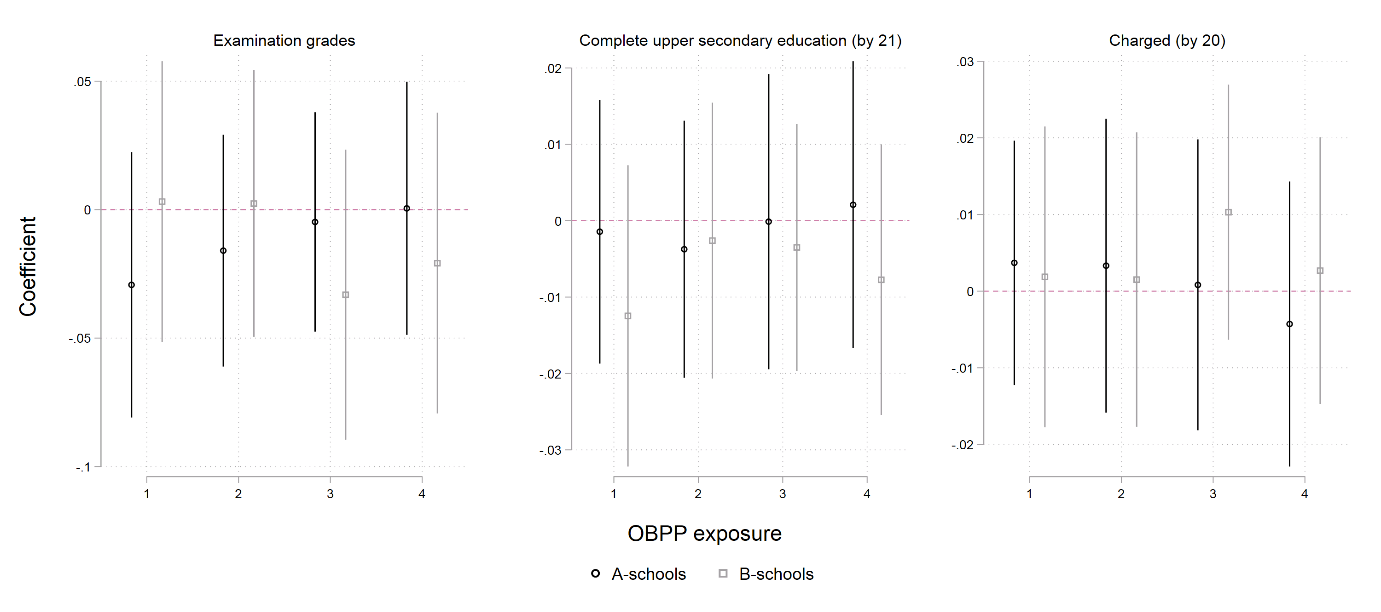


**Appendix Figure C11:** Effect estimates by A-schools and B-schools with 95% CI.

Note: See Appendix Figure C9 for pre-trend. Outcome metrics: Standardized for examination grades. Observed share for criminal charges and completion of upper secondary education.


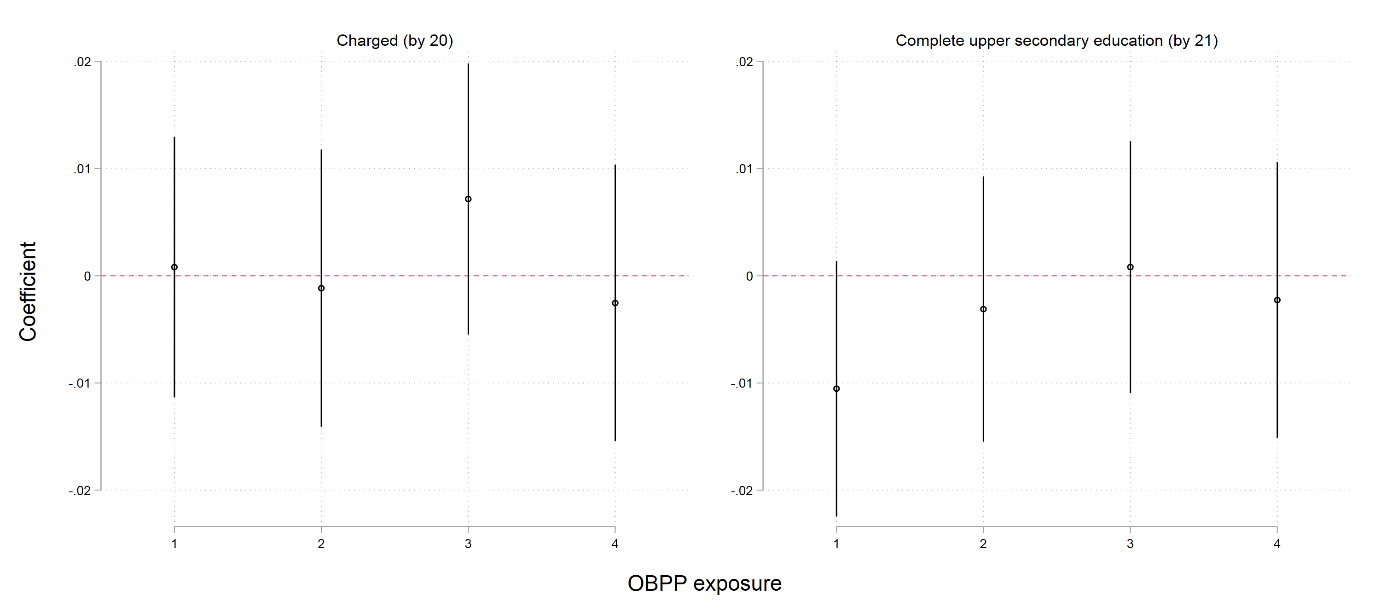


**Appendix Figure C12:** Average marginal effects from unconditional fixed effects logistic regressions with 95% CI.

Note: Outcome metrics: Observed share for criminal charges and completion of upper secondary education.

# Online Appendix D: Matched DiD

To identify program effects in the DiD setup, we need a group of comparison schools that serve as a counterfactual for what the development over time would have been had the treatment schools not implemented the OBPP program. In cases when the common trends assumption is not met, matching on pre-intervention levels may reduce bias (Ryan 2018; Ryan, Burgess Jr., and Dimick 2015). However, in cases where the common trends assumption does indeed hold, such matching may introduce bias because of regression to the mean (Daw and Hatfield 2018a). Thus, one should primarily use matched DiD when there are reasons to expect that all untreated cases constitute poor counterfactuals (Daw and Hatfield 2018b; Ryan 2018).

**Appendix Table D1:** Pre-intervention differences between schools that implement OBPP and control schools. Only pre-intervention cohorts were included (born 1986-1988).

|  | (1) | (2) |
| --- | --- | --- |
|  |  |  |
| **Panel I: Outcome variables** |  |  |
|  |  |  |
| Completed upp. sec. educ. (by 21) | 0.0116 | 0.0012 |
|  | (0.0077) | (0.0052) |
| Examination grades | 0.0461^*^ | 0.0143 |
|  | (0.0210) | (0.0120) |
| Charged (by 20) | -0.0040 | -0.0005 |
|  | (0.0050) | (0.0043) |
|  |  |  |
| **Panel II: Control variables** |  |  |
| Parents' education | 0.1081^*^ |  |
|  | (0.0506) |  |
| Parents' earnings (in 1000 NOK) | 11.7283 |  |
|  | (6.1898) |  |
| Immigrant background | 0.0154^*^ |  |
|  | (0.0075) |  |
|  |  |  |
| Control variables | No | Yes |

Note: Only pre-intervention cohorts were included (born 1986-1988). Standard errors clustered at the school level in parentheses.

^*^ *p* < 0.05, ^**^ *p* < 0.01, ^***^ *p* < 0.001

There are small differences between OBPP schools and control schools in both pre-intervention trends and pre-intervention levels in this study. As discussed above, inspecting leads and lags of program implementation (Appendix Figure C2) suggests that the concern of violation of common trends assumption is largely not warranted. Additionally, in the pre-intervention years, students who attend schools that will later implement OBPP have similar levels of grades, educational attainment, and charges than students who attend schools that will not implement OBPP (control schools) after accounting for observed control variables (Appendix Table D1, Appendix Figure D2).


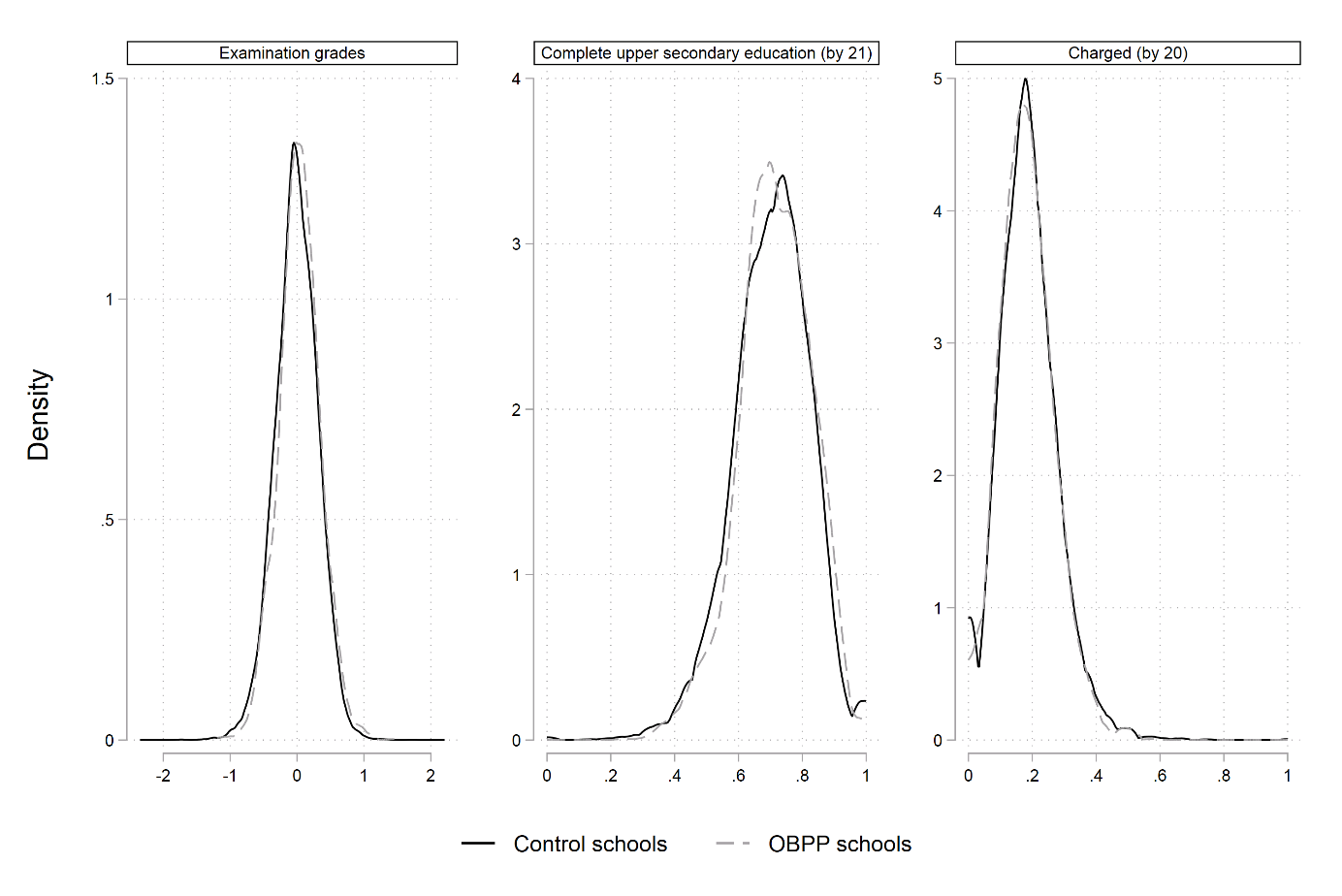


**Appendix Figure D1:** Descriptive differences at the school cohort between schools that implement OBPP and control schools in pre-intervention cohorts (born 1986-1988).

Nevertheless, because of the importance of the common trend assumption, we combine the DiD model with a matching algorithm to find control schools that are similar to the OBPP schools with regard to geographical proximity and pre-intervention level of the outcome variable as a final robustness check. More specifically, for each of the OBPP schools, we calculate the absolute difference in pre-intervention levels of the outcome between the OBPP schools and the pre-intervention levels in the corresponding birth cohorts in all control schools. We then match each of the OBPP schools with the control school within the school municipality with the lowest absolute difference in the pre-intervention level in the outcome variable, with the same control school potentially being a match for more than one OBPP school (matching with replacement). To avoid poor matches, however, we restrict possible matches to a maximum absolute difference of ½ standard deviation of the overall student-weighted school average of the outcome variable. If there are no suitable control schools within the school municipality, we look at matches in two of the neighboring municipalities.^[[3]](#footnote-3)^ We exclude about 15-20 percent of the OBPP schools because of no suitable matches within municipalities nor neighboring municipalities (Appendix Table D2). The results using a matched DiD model are similar to our main results (Appendix Figure D2).

**Appendix Table D2:** Number of schools matched on pre-intervention levels within municipality and neighboring municipality.

|  | Number of  schools matched | |  |
| --- | --- | --- | --- |
|  | Same municipality | Neighboring municipality |  |
|  |  |  |  |
| Charged (by 20) | 98 | 63 |  |
| Completed up. sec. educ. (by 21) | 102 | 75 |  |
| Examination grades | 102 | 68 |  |


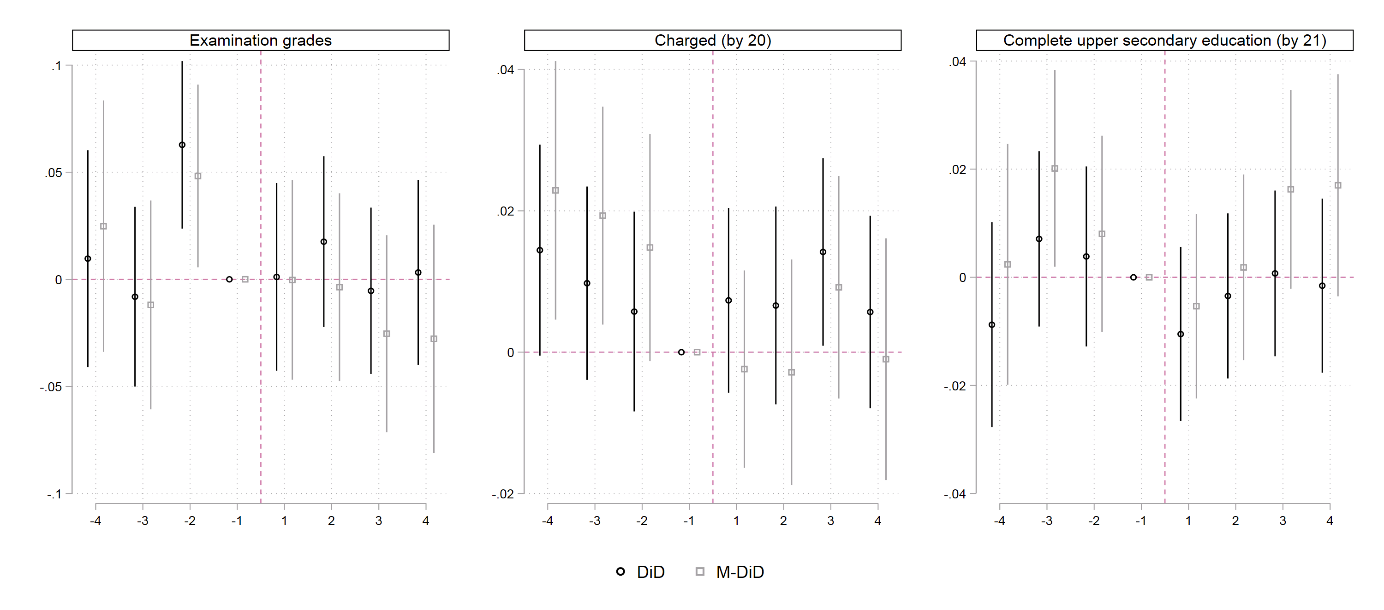


**Appendix Figure D2:** Effect estimates (after) and pre-program heterogeneity in a matched-DiD model with 95% CI.

Note: Outcome metrics: Standardized for examination grades. Observed share for criminal charges and completion of upper secondary education.

# Online Appendix References

Angrist, J. D., & Pischke, J.-S. (2009). *Mostly harmless econometrics: an empiricist's companion*. Princeton: Princeton University Press.

Cameron, A. C., & Miller, D. L. (2015). A practitioner’s guide to cluster-robust inference. *Journal of Human Resources, 50*(2), 317-372.

Daw, J. R., & Hatfield, L. A. (2018a). Matching and Regression to the Mean in Difference-in-Differences Analysis. *Health Services Research, 53*(6), 4138-4156.

Daw, J. R., & Hatfield, L. A. (2018b). Matching in Difference-in-Differences: between a Rock and a Hard Place. *Health Services Research, 53*(6), 4111-4117.

Lewbel, A. (2007). Estimation of average treatment effects with misclassification. *Econometrica, 75*(2), 537-551.

Mood, C. (2010). Logistic Regression: Why We Cannot Do What We Think We Can Do, and What We Can Do About It. *European Sociological Review, 26*(1), 67-82.

Ryan, A. M. (2018). Well-Balanced or too Matchy–Matchy? The Controversy over Matching in Difference-in-Differences. *Health Services Research, 53*(6), 4106-4110.

Ryan, A. M., Burgess Jr., J. F., & Dimick, J. B. (2015). Why We Should Not Be Indifferent to Specification Choices for Difference-in-Differences. *Health Services Research, 50*(4), 1211-1235.

1. Note that we do not need to assign students correctly to schools, but rather, to the correct OBPP treatment status. This means that we need to assign students to a school with the correct year of OBPP implementation. [↑](#footnote-ref-1)
2. We have used Stata 16.0 to run all analyses. [↑](#footnote-ref-2)
3. Neighboring municipalities is identified based on the official municipality number, where neighboring municipalities often have successive numbers. [↑](#footnote-ref-3)
